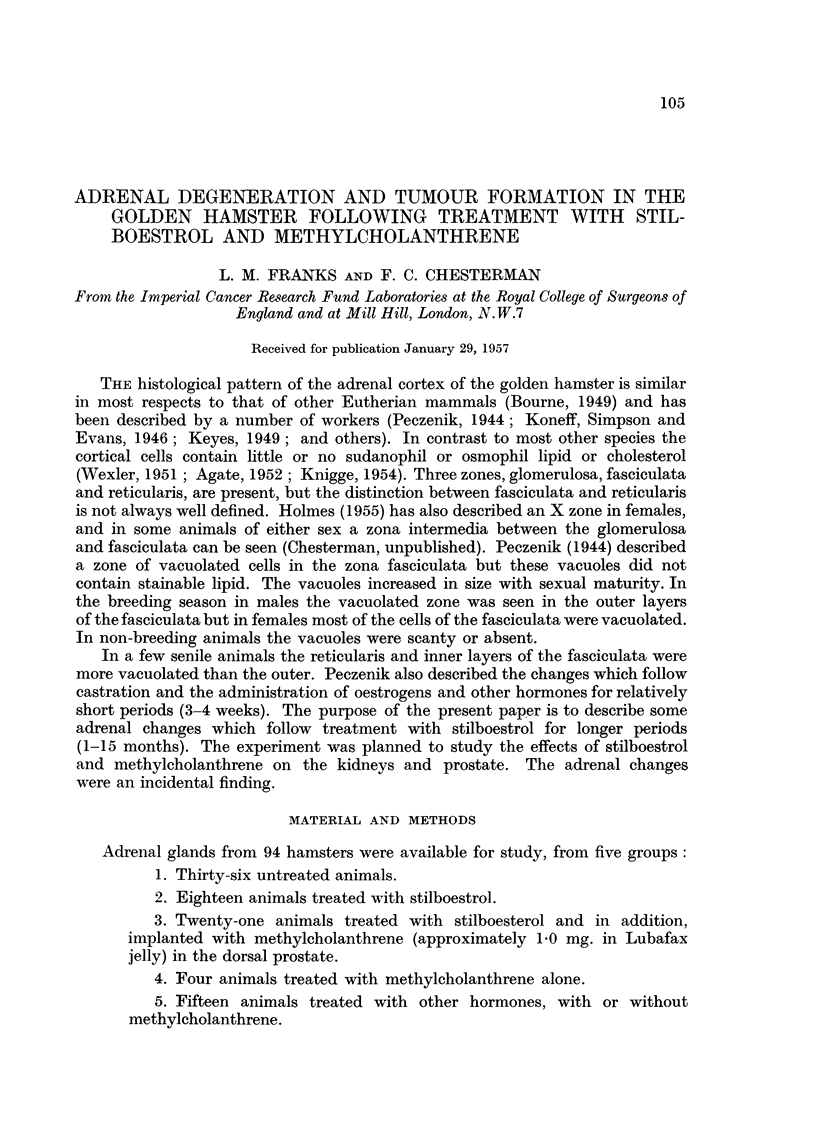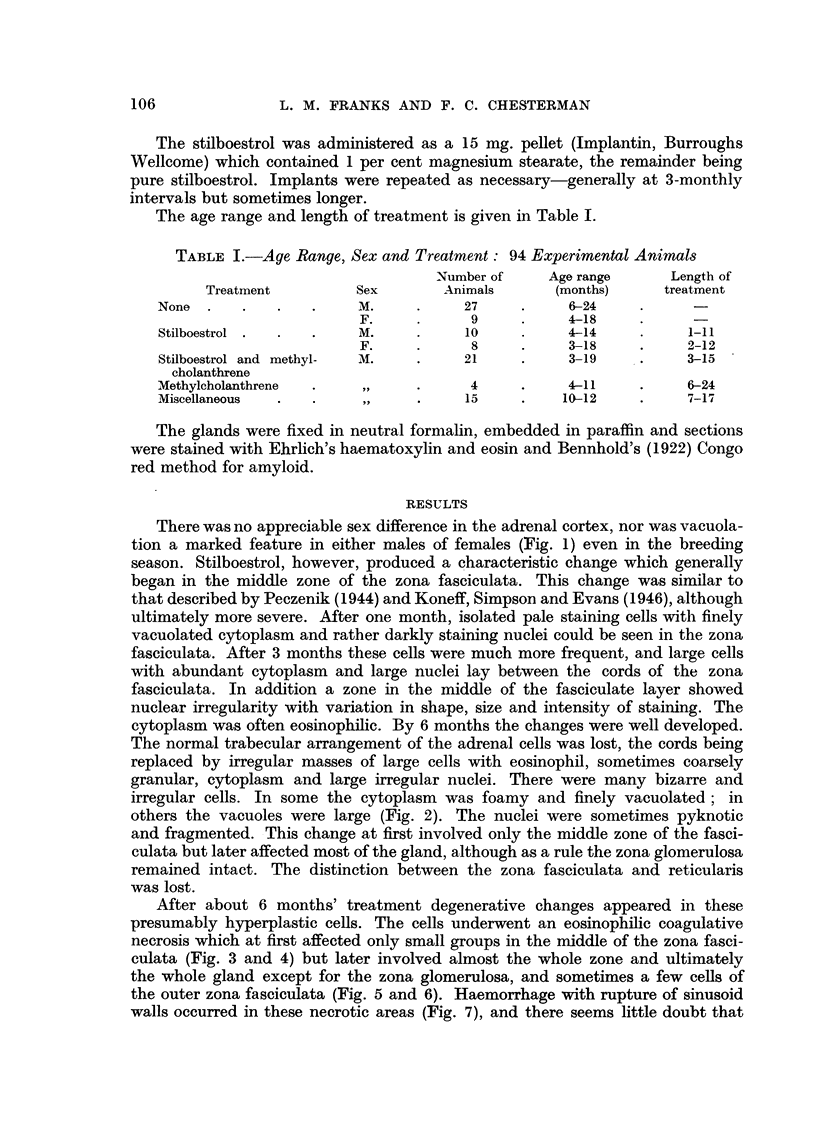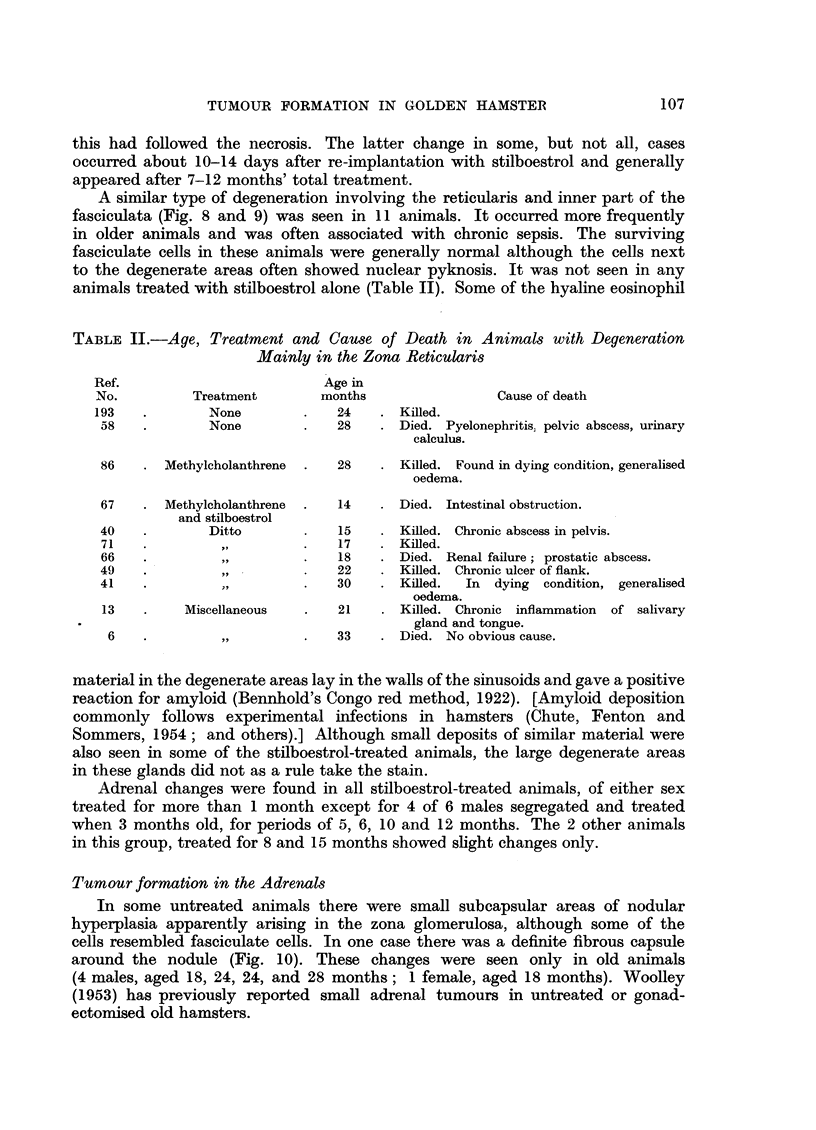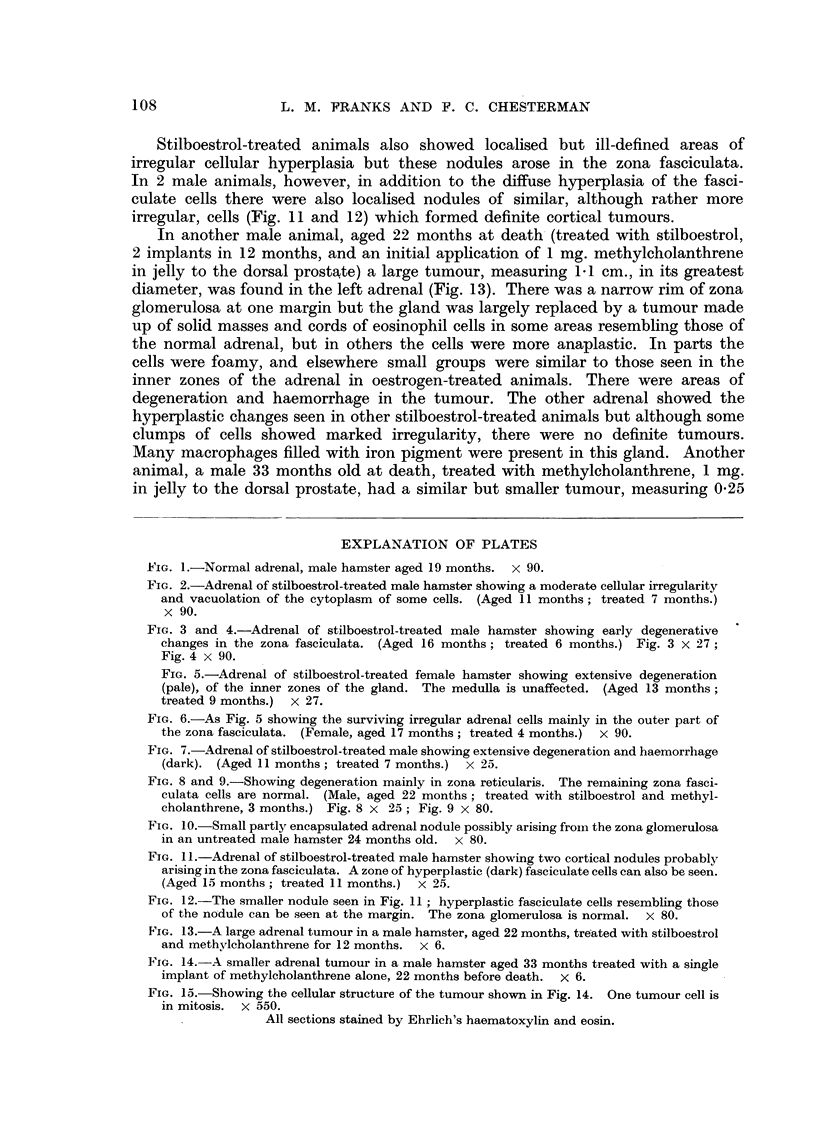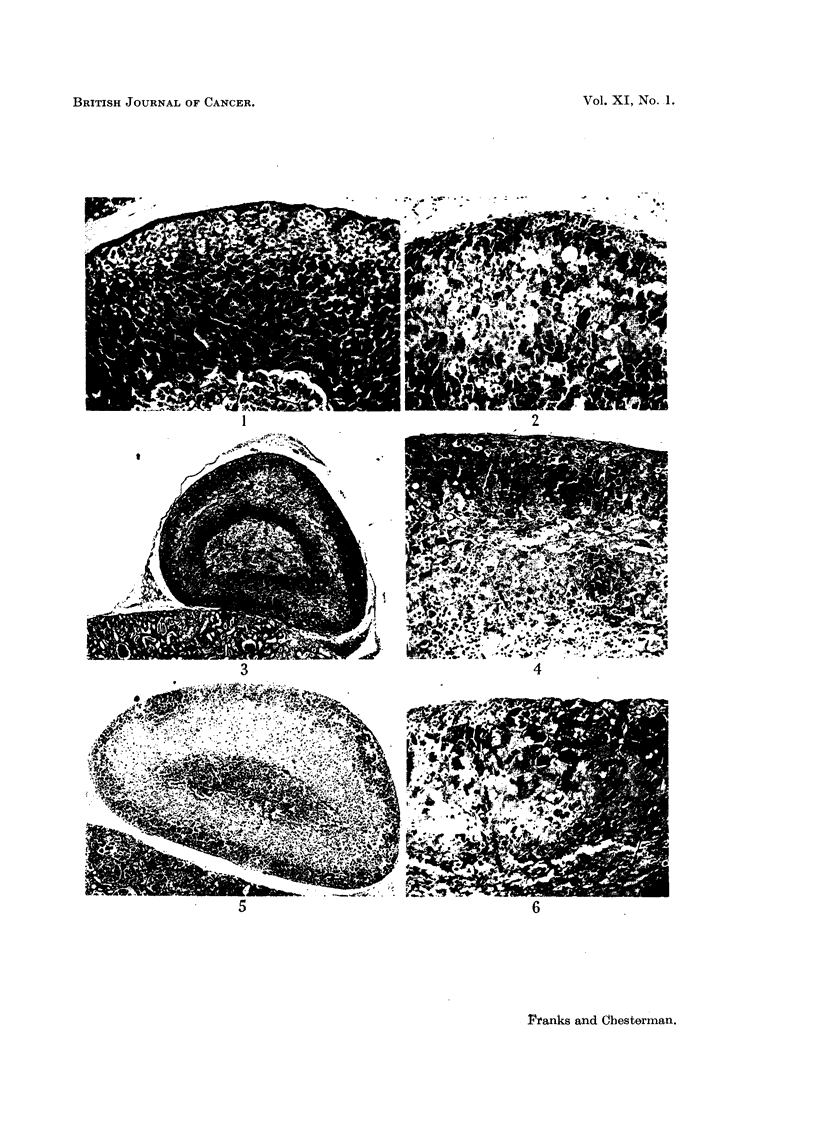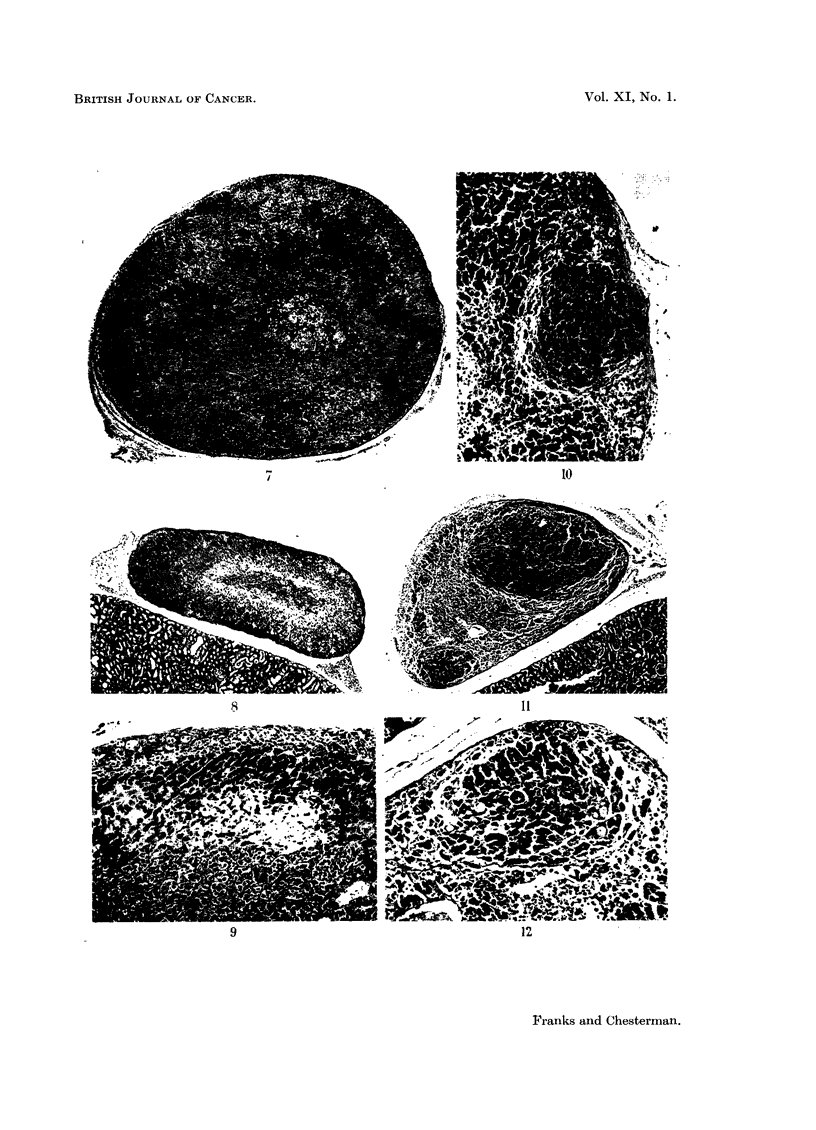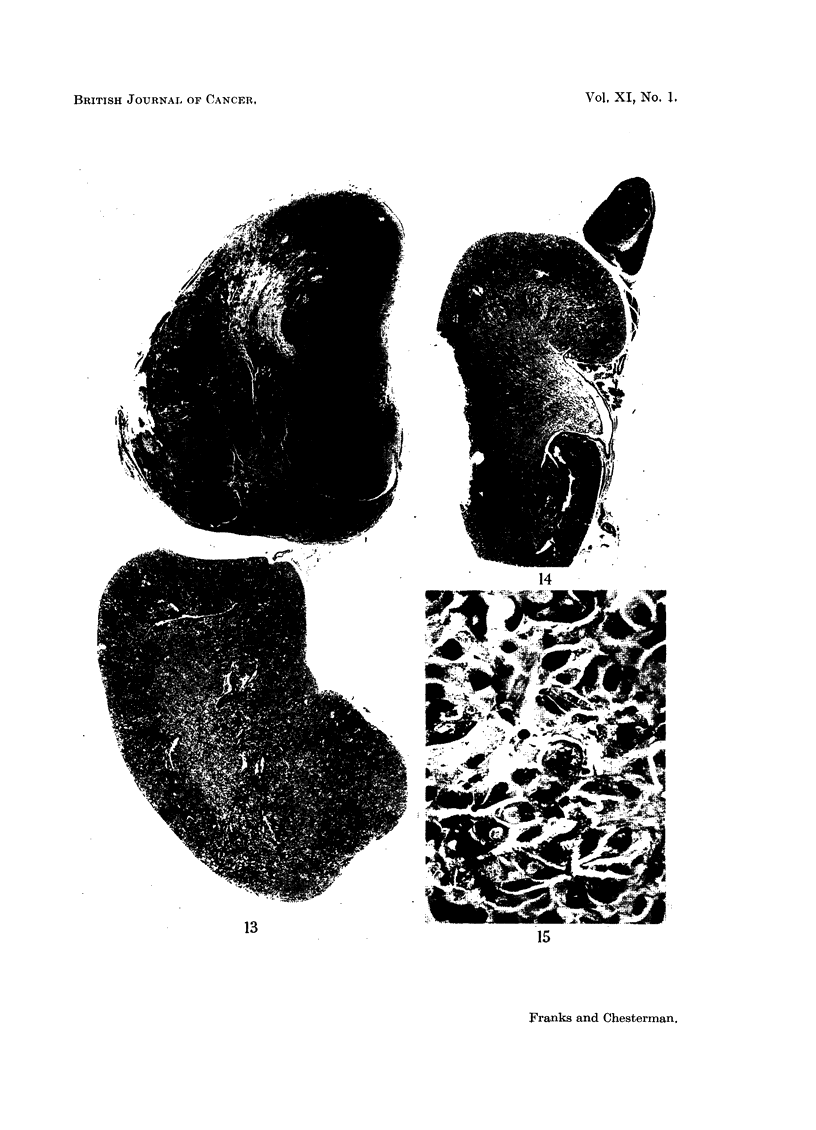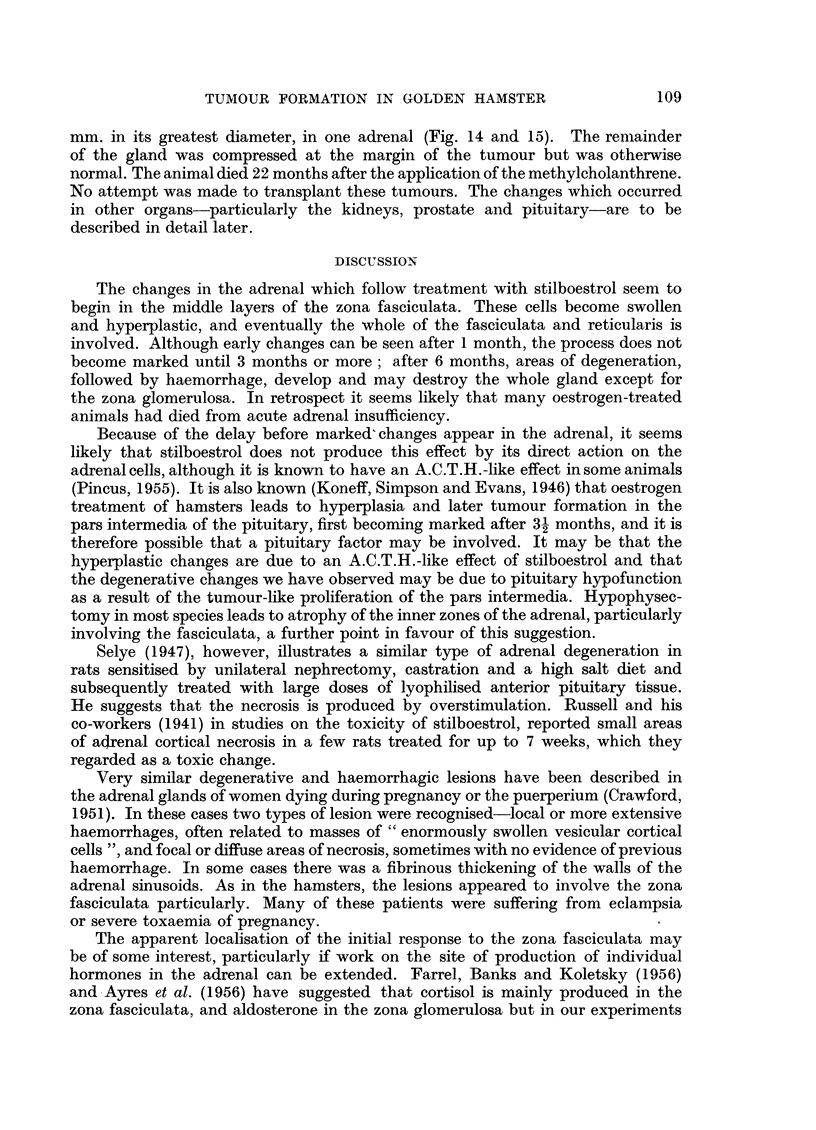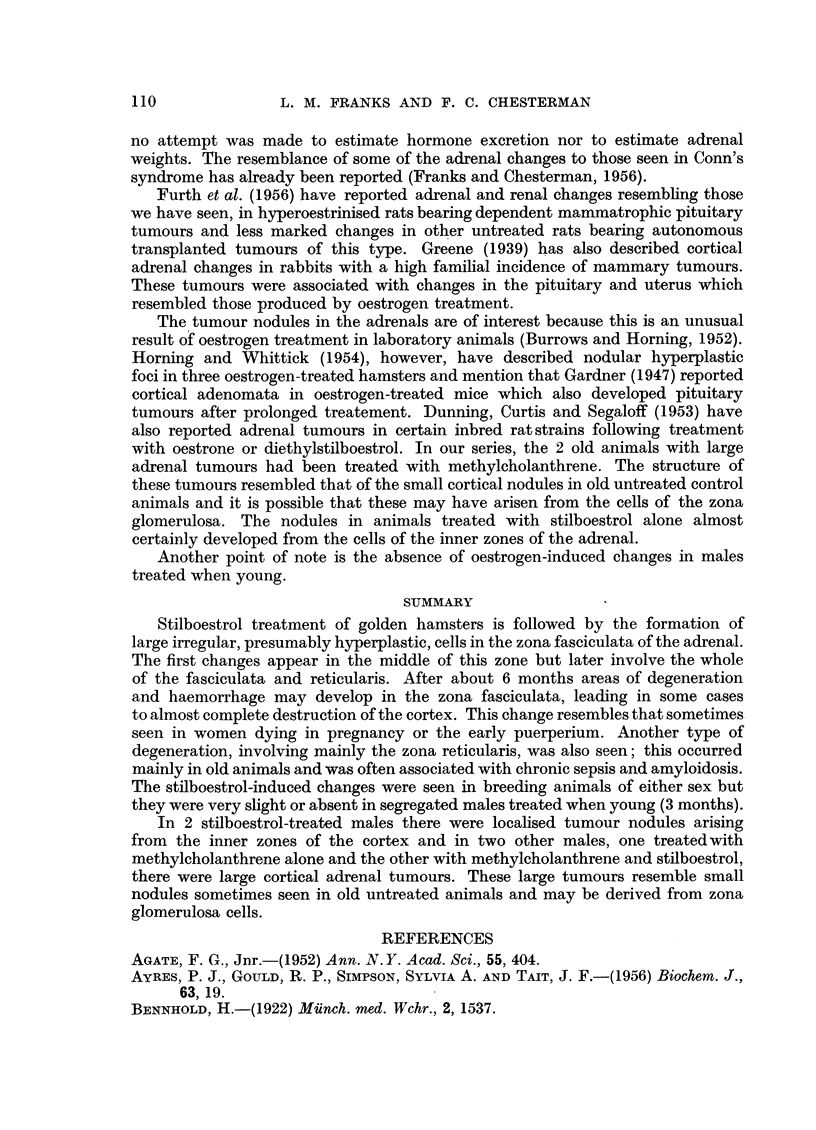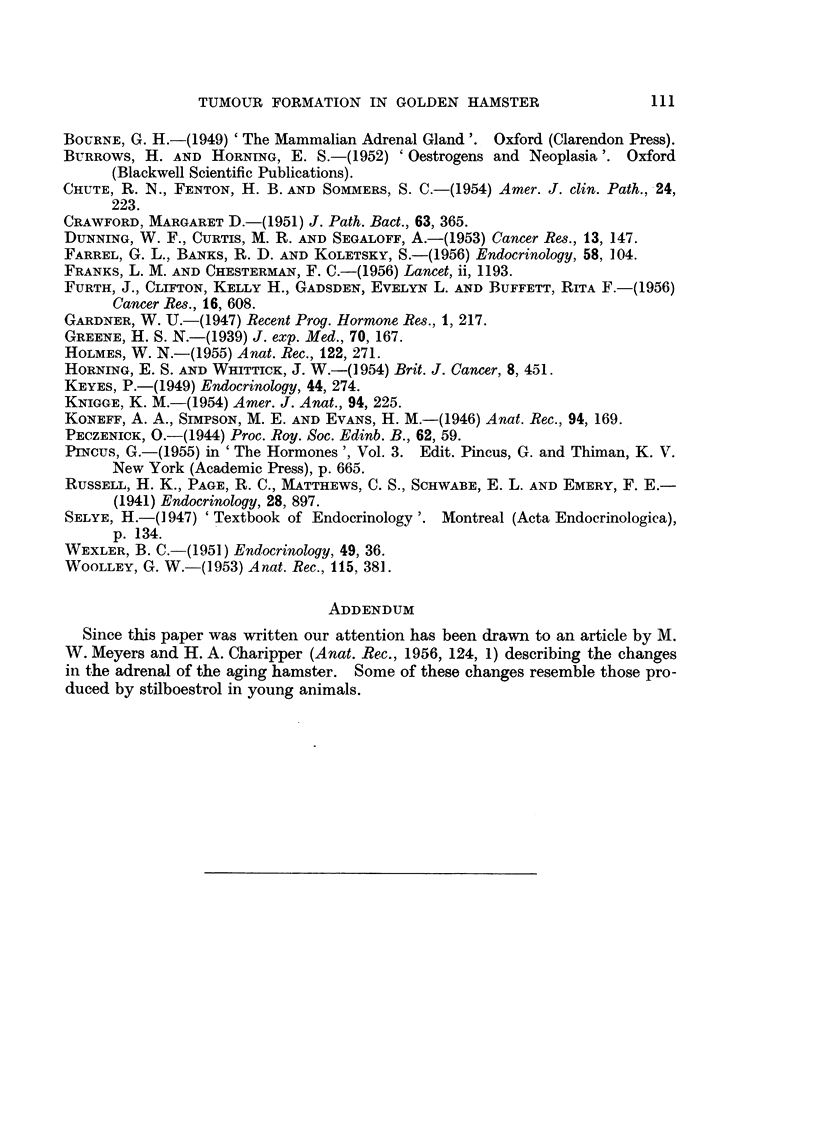# Adrenal Degeneration and Tumour Formation in the Golden Hamster Following Treatment with Stilboestrol and Methylcholanthrene

**DOI:** 10.1038/bjc.1957.15

**Published:** 1957-03

**Authors:** L. M. Franks, F. C. Chesterman

## Abstract

**Images:**


### ADRENAL DEGENERATION AND TUMOUR FORMATION IN THE GOLDEN HAMSTER FOLLOWING TREATMENT WITH STILBOESTROL AND METHYLCHOLANTHRENE

L. M. FRANKS AND F. C. CHESTERMAN

*From the Imperial Cancer Research Fund Laboratories at the Royal College of Surgeons of England and at Mill Hill, London, N.W.7*

Received for publication January 29, 1957

THE histological pattern of the adrenal cortex of the golden hamster is similar in most respects to that of other Eutherian mammals (Bourne, 1949) and has been described by a number of workers (Peczenik, 1944 ; Koneff, Simpson and Evans, 1946 ; Keyes, 1949 ; and others). In contrast to most other species the cortical cells contain little or no sudanophil or osmophil lipid or cholesterol (Wexler, 1951 ; Agate, 1952 ; Knigge, 1954). Three zones, glomerulosa, fasciculata and reticularis, are present, but the distinction between fasciculata and reticularis is not always well defined. Holmes (1955) has also described an X zone in females, and in some animals of either sex a zona intermedia between the glomerulosa and fasciculata can be seen (Chesterman, unpublished). Peczenik (1944) described a zone of vacuolated cells in the zona fasciculata but these vacuoles did not contain stainable lipid. The vacuoles increased in size with sexual maturity. In the breeding season in males the vacuolated zone was seen in the outer layers of the fasciculata but in females most of the cells of the fasciculata were vacuolated. In non-breeding animals the vacuoles were scanty or absent.

In a few senile animals the reticularis and inner layers of the fasciculata were more vacuolated than the outer. Peczenik also described the changes which follow castration and the administration of oestrogens and other hormones for relatively short periods (3–4 weeks). The purpose of the present paper is to describe some adrenal changes which follow treatment with stilboestrol for longer periods (1–15 months). The experiment was planned to study the effects of stilboestrol and methylcholanthrene on the kidneys and prostate. The adrenal changes were an incidental finding.

### MATERIAL AND METHODS

Adrenal glands from 94 hamsters were available for study, from five groups :

1. Thirty-six untreated animals.
2. Eighteen animals treated with stilboestrol.
3. Twenty-one animals treated with stilboesterol and in addition, implanted with methylcholanthrene (approximately 1·0 mg. in Lubafax jelly) in the dorsal prostate.
4. Four animals treated with methylcholanthrene alone.
5. Fifteen animals treated with other hormones, with or without methylcholanthrene.

The stilboestrol was administered as a 15 mg. pellet (Implantin, Burroughs Wellcome) which contained 1 per cent magnesium stearate, the remainder being pure stilboestrol. Implants were repeated as necessary—generally at 3-monthly intervals but sometimes longer.

The age range and length of treatment is given in Table I.

TABLE I.—*Age Range, Sex and Treatment : 94 Experimental Animals*

| Treatment | Sex | Number of Animals | Age range (months) | Length of treatment |
|---|---|---|---|---|
| None | M. | 27 | 6–24 | — |
| | F. | 9 | 4–18 | — |
| Stilboestrol | M. | 10 | 4–14 | 1–11 |
| | F. | 8 | 3–18 | 2–12 |
| Stilboestrol and methylcholanthrene | M. | 21 | 3–19 | 3–15 |
| Methylcholanthrene | ,, | 4 | 4–11 | 6–24 |
| Miscellaneous | ,, | 15 | 10–12 | 7–17 |

The glands were fixed in neutral formalin, embedded in paraffin and sections were stained with Ehrlich's haematoxylin and eosin and Bennhold's (1922) Congo red method for amyloid.

### RESULTS

There was no appreciable sex difference in the adrenal cortex, nor was vacuolation a marked feature in either males of females (Fig. 1) even in the breeding season. Stilboestrol, however, produced a characteristic change which generally began in the middle zone of the zona fasciculata. This change was similar to that described by Peczenik (1944) and Koneff, Simpson and Evans (1946), although ultimately more severe. After one month, isolated pale staining cells with finely vacuolated cytoplasm and rather darkly staining nuclei could be seen in the zona fasciculata. After 3 months these cells were much more frequent, and large cells with abundant cytoplasm and large nuclei lay between the cords of the zona fasciculata. In addition a zone in the middle of the fasciculate layer showed nuclear irregularity with variation in shape, size and intensity of staining. The cytoplasm was often eosinophilic. By 6 months the changes were well developed. The normal trabecular arrangement of the adrenal cells was lost, the cords being replaced by irregular masses of large cells with eosinophil, sometimes coarsely granular, cytoplasm and large irregular nuclei. There were many bizarre and irregular cells. In some the cytoplasm was foamy and finely vacuolated ; in others the vacuoles were large (Fig. 2). The nuclei were sometimes pyknotic and fragmented. This change at first involved only the middle zone of the fasciculata but later affected most of the gland, although as a rule the zona glomerulosa remained intact. The distinction between the zona fasciculata and reticularis was lost.

After about 6 months' treatment degenerative changes appeared in these presumably hyperplastic cells. The cells underwent an eosinophilic coagulative necrosis which at first affected only small groups in the middle of the zona fasciculata (Fig. 3 and 4) but later involved almost the whole zone and ultimately the whole gland except for the zona glomerulosa, and sometimes a few cells of the outer zona fasciculata (Fig. 5 and 6). Haemorrhage with rupture of sinusoid walls occurred in these necrotic areas (Fig. 7), and there seems little doubt that this had followed the necrosis. The latter change in some, but not all, cases occurred about 10–14 days after re-implantation with stilboestrol and generally appeared after 7–12 months' total treatment.

A similar type of degeneration involving the reticularis and inner part of the fasciculata (Fig. 8 and 9) was seen in 11 animals. It occurred more frequently in older animals and was often associated with chronic sepsis. The surviving fasciculate cells in these animals were generally normal although the cells next to the degenerate areas often showed nuclear pyknosis. It was not seen in any animals treated with stilboestrol alone (Table II). Some of the hyaline eosinophil

TABLE II.—*Age, Treatment and Cause of Death in Animals with Degeneration Mainly in the Zona Reticularis*

| Ref. No. | Treatment | Age in months | Cause of death |
|---|---|---|---|
| 193 | None | 24 | Killed. |
| 58 | None | 28 | Died. Pyelonephritis, pelvic abscess, urinary calculus. |
| 86 | Methylcholanthrene | 28 | Killed. Found in dying condition, generalised oedema. |
| 67 | Methylcholanthrene and stilboestrol | 14 | Died. Intestinal obstruction. |
| 40 | Ditto | 15 | Killed. Chronic abscess in pelvis. |
| 71 | ,, | 17 | Killed. |
| 66 | ,, | 18 | Died. Renal failure ; prostatic abscess. |
| 49 | ,, | 22 | Killed. Chronic ulcer of flank. |
| 41 | ,, | 30 | Killed. In dying condition, generalised oedema. |
| 13 | Miscellaneous | 21 | Killed. Chronic inflammation of salivary gland and tongue. |
| 6 | ,, | 33 | Died. No obvious cause. |

material in the degenerate areas lay in the walls of the sinusoids and gave a positive reaction for amyloid (Bennhold's Congo red method, 1922). [Amyloid deposition commonly follows experimental infections in hamsters (Chute, Fenton and Sommers, 1954 ; and others).] Although small deposits of similar material were also seen in some of the stilboestrol-treated animals, the large degenerate areas in these glands did not as a rule take the stain.

Adrenal changes were found in all stilboestrol-treated animals, of either sex treated for more than 1 month except for 4 of 6 males segregated and treated when 3 months old, for periods of 5, 6, 10 and 12 months. The 2 other animals in this group, treated for 8 and 15 months showed slight changes only.

*Tumour formation in the Adrenals*

In some untreated animals there were small subcapsular areas of nodular hyperplasia apparently arising in the zona glomerulosa, although some of the cells resembled fasciculate cells. In one case there was a definite fibrous capsule around the nodule (Fig. 10). These changes were seen only in old animals (4 males, aged 18, 24, 24, and 28 months ; 1 female, aged 18 months). Woolley (1953) has previously reported small adrenal tumours in untreated or gonadectomised old hamsters.

Stilboestrol-treated animals also showed localised but ill-defined areas of irregular cellular hyperplasia but these nodules arose in the zona fasciculata. In 2 male animals, however, in addition to the diffuse hyperplasia of the fasciculate cells there were also localised nodules of similar, although rather more irregular, cells (Fig. 11 and 12) which formed definite cortical tumours.

In another male animal, aged 22 months at death (treated with stilboestrol, 2 implants in 12 months, and an initial application of 1 mg. methylcholanthrene in jelly to the dorsal prostate) a large tumour, measuring 1·1 cm., in its greatest diameter, was found in the left adrenal (Fig. 13). There was a narrow rim of zona glomerulosa at one margin but the gland was largely replaced by a tumour made up of solid masses and cords of eosinophil cells in some areas resembling those of the normal adrenal, but in others the cells were more anaplastic. In parts the cells were foamy, and elsewhere small groups were similar to those seen in the inner zones of the adrenal in oestrogen-treated animals. There were areas of degeneration and haemorrhage in the tumour. The other adrenal showed the hyperplastic changes seen in other stilboestrol-treated animals but although some clumps of cells showed marked irregularity, there were no definite tumours. Many macrophages filled with iron pigment were present in this gland. Another animal, a male 33 months old at death, treated with methylcholanthrene, 1 mg. in jelly to the dorsal prostate, had a similar but smaller tumour, measuring 0·25

---

EXPLANATION OF PLATES

FIG. 1.—Normal adrenal, male hamster aged 19 months. × 90.

FIG. 2.—Adrenal of stilboestrol-treated male hamster showing a moderate cellular irregularity and vacuolation of the cytoplasm of some cells. (Aged 11 months ; treated 7 months.) × 90.

FIG. 3 and 4.—Adrenal of stilboestrol-treated male hamster showing early degenerative changes in the zona fasciculata. (Aged 16 months ; treated 6 months.) Fig. 3 × 27 ; Fig. 4 × 90.

FIG. 5.—Adrenal of stilboestrol-treated female hamster showing extensive degeneration (pale), of the inner zones of the gland. The medulla is unaffected. (Aged 13 months ; treated 9 months.) × 27.

FIG. 6.—As Fig. 5 showing the surviving irregular adrenal cells mainly in the outer part of the zona fasciculata. (Female, aged 17 months ; treated 4 months.) × 90.

FIG. 7.—Adrenal of stilboestrol-treated male showing extensive degeneration and haemorrhage (dark). (Aged 11 months ; treated 7 months.) × 25.

FIG. 8 and 9.—Showing degeneration mainly in zona reticularis. The remaining zona fasciculata cells are normal. (Male, aged 22 months ; treated with stilboestrol and methylcholanthrene, 3 months.) Fig. 8 × 25 ; Fig. 9 × 80.

FIG. 10.—Small partly encapsulated adrenal nodule possibly arising from the zona glomerulosa in an untreated male hamster 24 months old. × 80.

FIG. 11.—Adrenal of stilboestrol-treated male hamster showing two cortical nodules probably arising in the zona fasciculata. A zone of hyperplastic (dark) fasciculate cells can also be seen. (Aged 15 months ; treated 11 months.) × 25.

FIG. 12.—The smaller nodule seen in Fig. 11 ; hyperplastic fasciculate cells resembling those of the nodule can be seen at the margin. The zona glomerulosa is normal. × 80.

FIG. 13.—A large adrenal tumour in a male hamster, aged 22 months, treated with stilboestrol and methylcholanthrene for 12 months. × 6.

FIG. 14.—A smaller adrenal tumour in a male hamster aged 33 months treated with a single implant of methylcholanthrene alone, 22 months before death. × 6.

FIG. 15.—Showing the cellular structure of the tumour shown in Fig. 14. One tumour cell is in mitosis. × 550.

All sections stained by Ehrlich's haematoxylin and eosin.

1 2 3 4 5 6

7
10
8
11
9
12

13

14

15

Franks and Chesterman.

mm. in its greatest diameter, in one adrenal (Fig. 14 and 15). The remainder of the gland was compressed at the margin of the tumour but was otherwise normal. The animal died 22 months after the application of the methylcholanthrene. No attempt was made to transplant these tumours. The changes which occurred in other organs—particularly the kidneys, prostate and pituitary—are to be described in detail later.

### DISCUSSION

The changes in the adrenal which follow treatment with stilboestrol seem to begin in the middle layers of the zona fasciculata. These cells become swollen and hyperplastic, and eventually the whole of the fasciculata and reticularis is involved. Although early changes can be seen after 1 month, the process does not become marked until 3 months or more ; after 6 months, areas of degeneration, followed by haemorrhage, develop and may destroy the whole gland except for the zona glomerulosa. In retrospect it seems likely that many oestrogen-treated animals had died from acute adrenal insufficiency.

Because of the delay before marked changes appear in the adrenal, it seems likely that stilboestrol does not produce this effect by its direct action on the adrenal cells, although it is known to have an A.C.T.H.-like effect in some animals (Pincus, 1955). It is also known (Koneff, Simpson and Evans, 1946) that oestrogen treatment of hamsters leads to hyperplasia and later tumour formation in the pars intermedia of the pituitary, first becoming marked after $3\frac{1}{2}$ months, and it is therefore possible that a pituitary factor may be involved. It may be that the hyperplastic changes are due to an A.C.T.H.-like effect of stilboestrol and that the degenerative changes we have observed may be due to pituitary hypofunction as a result of the tumour-like proliferation of the pars intermedia. Hypophysectomy in most species leads to atrophy of the inner zones of the adrenal, particularly involving the fasciculata, a further point in favour of this suggestion.

Selye (1947), however, illustrates a similar type of adrenal degeneration in rats sensitised by unilateral nephrectomy, castration and a high salt diet and subsequently treated with large doses of lyophilised anterior pituitary tissue. He suggests that the necrosis is produced by overstimulation. Russell and his co-workers (1941) in studies on the toxicity of stilboestrol, reported small areas of adrenal cortical necrosis in a few rats treated for up to 7 weeks, which they regarded as a toxic change.

Very similar degenerative and haemorrhagic lesions have been described in the adrenal glands of women dying during pregnancy or the puerperium (Crawford, 1951). In these cases two types of lesion were recognised—local or more extensive haemorrhages, often related to masses of " enormously swollen vesicular cortical cells ", and focal or diffuse areas of necrosis, sometimes with no evidence of previous haemorrhage. In some cases there was a fibrinous thickening of the walls of the adrenal sinusoids. As in the hamsters, the lesions appeared to involve the zona fasciculata particularly. Many of these patients were suffering from eclampsia or severe toxaemia of pregnancy.

The apparent localisation of the initial response to the zona fasciculata may be of some interest, particularly if work on the site of production of individual hormones in the adrenal can be extended. Farrel, Banks and Koletsky (1956) and Ayres *et al.* (1956) have suggested that cortisol is mainly produced in the zona fasciculata, and aldosterone in the zona glomerulosa but in our experiments no attempt was made to estimate hormone excretion nor to estimate adrenal weights. The resemblance of some of the adrenal changes to those seen in Conn's syndrome has already been reported (Franks and Chesterman, 1956).

Furth *et al.* (1956) have reported adrenal and renal changes resembling those we have seen, in hyperoestrinised rats bearing dependent mammatrophic pituitary tumours and less marked changes in other untreated rats bearing autonomous transplanted tumours of this type. Greene (1939) has also described cortical adrenal changes in rabbits with a high familial incidence of mammary tumours. These tumours were associated with changes in the pituitary and uterus which resembled those produced by oestrogen treatment.

The tumour nodules in the adrenals are of interest because this is an unusual result of oestrogen treatment in laboratory animals (Burrows and Horning, 1952). Horning and Whittick (1954), however, have described nodular hyperplastic foci in three oestrogen-treated hamsters and mention that Gardner (1947) reported cortical adenomata in oestrogen-treated mice which also developed pituitary tumours after prolonged treatement. Dunning, Curtis and Segaloff (1953) have also reported adrenal tumours in certain inbred rat strains following treatment with oestrone or diethylstilboestrol. In our series, the 2 old animals with large adrenal tumours had been treated with methylcholanthrene. The structure of these tumours resembled that of the small cortical nodules in old untreated control animals and it is possible that these may have arisen from the cells of the zona glomerulosa. The nodules in animals treated with stilboestrol alone almost certainly developed from the cells of the inner zones of the adrenal.

Another point of note is the absence of oestrogen-induced changes in males treated when young.

### SUMMARY

Stilboestrol treatment of golden hamsters is followed by the formation of large irregular, presumably hyperplastic, cells in the zona fasciculata of the adrenal. The first changes appear in the middle of this zone but later involve the whole of the fasciculata and reticularis. After about 6 months areas of degeneration and haemorrhage may develop in the zona fasciculata, leading in some cases to almost complete destruction of the cortex. This change resembles that sometimes seen in women dying in pregnancy or the early puerperium. Another type of degeneration, involving mainly the zona reticularis, was also seen; this occurred mainly in old animals and was often associated with chronic sepsis and amyloidosis. The stilboestrol-induced changes were seen in breeding animals of either sex but they were very slight or absent in segregated males treated when young (3 months).

In 2 stilboestrol-treated males there were localised tumour nodules arising from the inner zones of the cortex and in two other males, one treated with methylcholanthrene alone and the other with methylcholanthrene and stilboestrol, there were large cortical adrenal tumours. These large tumours resemble small nodules sometimes seen in old untreated animals and may be derived from zona glomerulosa cells.

### REFERENCES

AGATE, F. G., Jnr.—(1952) *Ann. N.Y. Acad. Sci.*, **55**, 404.

AYRES, P. J., GOULD, R. P., SIMPSON, SYLVIA A. AND TAIT, J. F.—(1956) *Biochem. J.*, **63**, 19.

BENNHOLD, H.—(1922) *Münch. med. Wchr.*, **2**, 1537.

BOURNE, G. H.—(1949) 'The Mammalian Adrenal Gland'. Oxford (Clarendon Press).
BURROWS, H. AND HORNING, E. S.—(1952) 'Oestrogens and Neoplasia'. Oxford (Blackwell Scientific Publications).
CHUTE, R. N., FENTON, H. B. AND SOMMERS, S. C.—(1954) *Amer. J. clin. Path.*, **24**, 223.
CRAWFORD, MARGARET D.—(1951) *J. Path. Bact.*, **63**, 365.
DUNNING, W. F., CURTIS, M. R. AND SEGALOFF, A.—(1953) *Cancer Res.*, **13**, 147.
FARREL, G. L., BANKS, R. D. AND KOLETSKY, S.—(1956) *Endocrinology*, **58**, 104.
FRANKS, L. M. AND CHESTERMAN, F. C.—(1956) *Lancet*, ii, 1193.
FURTH, J., CLIFTON, KELLY H., GADSDEN, EVELYN L. AND BUFFETT, RITA F.—(1956) *Cancer Res.*, **16**, 608.
GARDNER, W. U.—(1947) *Recent Prog. Hormone Res.*, **1**, 217.
GREENE, H. S. N.—(1939) *J. exp. Med.*, **70**, 167.
HOLMES, W. N.—(1955) *Anat. Rec.*, **122**, 271.
HORNING, E. S. AND WHITTICK, J. W.—(1954) *Brit. J. Cancer*, **8**, 451.
KEYES, P.—(1949) *Endocrinology*, **44**, 274.
KNIGGE, K. M.—(1954) *Amer. J. Anat.*, **94**, 225.
KONEFF, A. A., SIMPSON, M. E. AND EVANS, H. M.—(1946) *Anat. Rec.*, **94**, 169.
PECZENICK, O.—(1944) *Proc. Roy. Soc. Edinb. B.*, **62**, 59.
PINCUS, G.—(1955) in 'The Hormones', Vol. 3. Edit. Pincus, G. and Thiman, K. V. New York (Academic Press), p. 665.
RUSSELL, H. K., PAGE, R. C., MATTHEWS, C. S., SCHWABE, E. L. AND EMERY, F. E.—(1941) *Endocrinology*, **28**, 897.
SELYE, H.—(1947) 'Textbook of Endocrinology'. Montreal (Acta Endocrinologica), p. 134.
WEXLER, B. C.—(1951) *Endocrinology*, **49**, 36.
WOOLLEY, G. W.—(1953) *Anat. Rec.*, **115**, 381.

### ADDENDUM

Since this paper was written our attention has been drawn to an article by M. W. Meyers and H. A. Charipper (*Anat. Rec.*, 1956, 124, 1) describing the changes in the adrenal of the aging hamster. Some of these changes resemble those produced by stilboestrol in young animals.